# Psychological distress among Italians during the 2019 coronavirus disease (COVID-19) quarantine

**DOI:** 10.1186/s12888-020-03027-8

**Published:** 2021-01-08

**Authors:** Maurizio Bonati, Rita Campi, Michele Zanetti, Massimo Cartabia, Francesca Scarpellini, Antonio Clavenna, Giulia Segre

**Affiliations:** Department of Public Health, Laboratory for Mother and Child Health, Istituto di Ricerche Farmacologiche Mario Negri IRCCS, Via Mario Negri 2, 20156 Milan, Italy

**Keywords:** Online surveys, Perceptions, Knowledge, Coronavirus, SARS-CoV-2, Pandemic, Infectious diseases, Outbreak, COVID-19, Public health

## Abstract

**Background:**

Quarantine as a preventive action to reduce people’s exposure to a contagious disease has substantial psychological impact. We aimed to collect information on psychologically distressing experiences of Italians living in quarantine during the COVID-19 pandemic.

**Methods:**

From 6 to 20 April 2020 participants filled out an online questionnaire. Demographic and physical symptoms data from the prior 14 days of quarantine were collected. Psychological impact of quarantine was assessed by the COVID-19 Peritraumatic Distress Index (CPDI).

**Results:**

In all, 20,158 participants completed the online survey. Of these, 11,910 (59.1%) were from Lombardy, the region with 37.7% of positive cases identified during the survey period. 30.1% of responders were male. About half (55.9%) of responders were 18–50 years old, 54.3% had a tertiary level of education, 69.5% were workers, 84.1% were living in houses with ≥3 rooms, and 13.7% were living alone. 9.7% had had contact with COVID-19 positive people. Of all responders, 9978 (48.6%) reported a psychological impact, 8897 (43.4%) of whom reported mild or moderate and 1081 (5.2%) severe psychological impact. The multivariate analysis, after adjustments, showed that an increasing CPDI score was associated with gender (female), first-second educational level, being unemployed, living in a ≤2 room house, having had new health problems during the previous 14 days, and not having been out of the house in the previous week. Concerning the type of psychological distress, 2003 responders (9.9%) reported moderate to severe depressive symptoms, 1131 (5.5%) moderate to severe anxiety symptoms, and 802 (3.9%) moderate to severe physical symptoms. A positive correlation was found between responder rate (per 10.000 residents) and positive COVID-19 cases (per 10.000 residents) by region (r_s_ = + 0.83, *p =* < 0.0001), and between responder rate and region latitude (r_s_ = + 0.91, *p =* < 0.0001), with a greater response rate in the north. Considering Lombardy Region responders, a negative correlation between CPDI score and distance from place of residence to the red zone (Nembro-Alzano) was found. Higher prevalence of psychological distress was found up to 25 km away from the red zone and, in particular, severe distress up to 15 km.

**Conclusions:**

Policy makers and mental health professionals should be aware of quarantine’s adverse mental health consequences. Factors influencing the success of quarantine and infection control practices for both disease containment and community recovery should be identified and additional support to vulnerable persons at increased risk of adverse psychological and social consequences of quarantine should be guaranteed.

**Supplementary Information:**

The online version contains supplementary material available at 10.1186/s12888-020-03027-8.

## Background

Since first being recorded in late 2019 in Wuhan, China, the severe acute respiratory syndrome (SARS)-coronavirus-2 (CoV-2) outbreak has spread around the world with an increasing death toll, and was declared a pandemic by the World Health Organization [1]. The disease has hit certain countries, including Italy, with particular cruelty. About 5 months after the start of the pandemic the number of suspected coronavirus disease 2019 (COVID-19) related deaths were 30,000 in Italy (of which half in the Lombardy Region), close to the number in the UK and half of the US estimates.

The spread of the virus is characterized by human-to-human transmission [2], and cannot be prevented by simply wearing facial masks. The only way to control this disease is to cut off the route of transmission, and home isolation is appropriate for preventing the spread of COVID-19 [3, 4]. Countries around the world implemented measures to slow the spread of the coronavirus, from national quarantines to school closures. Uncertainty on the characteristics of the COVID-19 pandemic reigned again after a few months from the beginning, especially aspects involving the mode and circumstances of transmission of this newly identified agent. One of the main uncertainties concerns the means by which COVID-19 is transmitted, with special regard to the factors that may accelerate or delay its spread, the mode of transmission, the role of asymptomatic infected people, its speed, the possible interactions with wildlife or livestock, urban or rural environments, and population density. Such uncertainties promoted extreme actions, such as the total lockdown of entire countries [5]. Very different approaches have been followed within and between countries, both in the types of interventions and in the implementation times. An estimated 2.6 billion people – one-third of the world’s population – lived for an extended period, even a couple of months, under some kind of lockdown or quarantine. From March 9th to May 5th Italy imposed the most restrictive measures of lockdown after those taken in Wuhan.

Quarantine is defined as the separation and restriction of movement of people who have potentially been exposed to a contagious disease to ascertain if they become unwell, thus reducing the risk of them infecting others [6].

Two recent reviews [7, 8] found severe mental health problems among individuals and populations who have undergone quarantine and isolation in different contexts. Some concerns were described as motivating agents for physical and emotional exhaustion, for example: the duration of this confinement, frustration, boredom, financial losses, social stigma, and inadequate receipt of supplies and information [7].

Social distancing and self-isolation during the COVID-19 pandemic further challenged the mental health and general wellbeing of people, contributing to increased interpersonal and intrapersonal issues such as domestic violence, family disfunction, and poor health outcomes [9]. Prevalent disorders among individuals included depression, anxiety, mood disorders, psychological distress, posttraumatic stress disorder, insomnia, fear, stigmatization, low self-esteem, lack of self-control, and other adverse mental health outcomes. In particular, some groups may be more vulnerable to the psychosocial effects of pandemics: people who contract the disease and their families, subjects at heightened risk of contracting it, people with pre-existing physical or psychological conditions, and health care workers [10]. A few studies have highlighted the psychological impact of the COVID-19 lockdown on the global population. Findings from a Chinese survey have shown that almost 35% of the participants experienced peritraumatic distress [11]. Similarly, another study conducted among the general Chinese population stated that 53.8% of respondents rated the psychological impact of the outbreak as moderate or severe. In particular, moderate to severe depressive symptoms were found in 16.5% of the sample; for the anxiety subscale, 63.3% were considered to have a normal score, meaning that nearly one third of the respondents reported moderate to severe anxiety symptoms [12].

In Europe, Italy was the first country to enter a nationwide lockdown and, to date, few studies have analyzed the effects of home confinement. A web-based survey assessed the mental health status of the general Italian population in the lockdown period: it showed post-traumatic stress in 37.1% of respondents, anxiety symptoms in 20.8%, severe depressive symptoms in 17.3%, and insomnia in 7.3% [13]. Considering factors that might predict mental health, studies showed a greater psychological impact of the outbreak on females, and on youths [11–13].

The present study was carried out with the following objectives:

1. to evaluate the impact of COVID-19 quarantine in Italy and to assess its effects on mental health and psychological wellbeing. In particular, anxiety, depressive, and physical symptoms were investigated among the Italian general population.
2. to explore potential factors, such as age and gender, that may contribute to, or mitigate, the effects on the mental health burden.
3. to investigate if in Lombardy, the most affected Italian region, there is a relationship between COVID-19 Peritraumatic Distress Index (CPDI) score and distance from place of residence to the red zone (Alzano Lombardo and Nembro municipalities).

## Methods

### Participants and procedures

This was a cross-sectional, study carried out in Italy. A dedicated website was created for the purpose of this study. An online, semi-structured questionnaire was developed by using Wordpress, a free open-source content management system (CMS), integrated with SurveyJS (survey library and survey creator), a library to facilitate survey creation and management. The survey script was available for all devices. The questionnaire used (Additional Table 1) was similar to those used in China during the tumultuous time of the COVID-19 epidemic [11], and to small surveys in Saudi Arabia [14], India [15], Iran [16], Brazil [17], Nepal [18], and Germany [19]. The socio-demographic section was modified to be more compliant with the Italian situation.

The questionnaire, which was validated in Italy [20], incorporated relevant diagnostic guidelines for specific phobias and stress disorders specified in the International Classification of Diseases, 11th Revision, and expert opinions from psychiatrists. The latter were necessary to define CPDI value inquiring about the frequency of anxiety, depression, specific phobias, cognitive change, avoidance and compulsive behaviour, physical symptoms, and loss of social functioning in the previous 2 weeks. A 5-point Likert-type scale was employed for all 24 items of the CPDI, with higher scores indicating higher distress: 0 “never”, 1 “occasionally”, 2 “sometimes”, 3 “often”, 4 “most of the time”. Raw score + 4 was the final CPDI score calculated for each participant. The scores ranged from 0 to 100. A score between 28 and 51 indicated mild to moderate distress. A score ≥52 indicated severe distress [11]. The Cronbach’s alpha reliability value was 0.895.

A snowball sampling technique was used. The link to the questionnaire, in Italian, was sent via e-mail, WhatsApp, and other social media to the contacts of the investigators. The participants, aged more than 18 years, were encouraged to roll out the survey to as many people as possible. The link was thus forwarded to an exponential number of people. To increase involvement, the email was sent to different mailing lists of people with whom the institute is in contact. When clicking on the link, the participants were directed to information on the study and to the informed consent form. After they accepted to take the survey they provided socio-demographic details including age, gender, occupation, education, and city and area of residence. Additionally, information on health problems during the previous 14 days, contact with COVID-19 positive people, and trips outside the house in the previous week were collected. A set of questions from the CPDI appeared sequentially, which the participants were asked to answer. The data collection was initiated on 6th April 2020 at 9 PM and closed on 20th April 2020 at 9 PM. We were able to collect data from all 20 Italian regions.

### Survey data analysis

The survey was classified as complete if all the questions related to the CPDI were answered.

Exploratory data analysis was conducted using frequency distributions for categorical variables, summarized using proportions, and associations were tested using chi-square or Fisher’s exact test where applicable. Continuous variables were summarized using means and standard deviations for normally distributed data, while skewed data were summarized using medians. One-way ANOVA (F-value) was used to test difference of means for normally distributed continuous variables and the Mann–Whitney U test for skewed continuous variables. We used Spearman’s correlation to determine the relationship between responder rate (per 10.000 residents) and positive COVID-19 cases (per 10.000 residents) by region at mid-survey. In the bivariate analyses, to identify factors influencing psychological distress (the combination of mild-moderate + severe), we computed odds ratios (OR) considering the significance of the confidence intervals (CI). In the multivariable analysis, a log-binomial regression model was used. All variables were entered into the model and a stepwise regression analysis was conducted. The Hosmer-Lemesshow test was used to determine the goodness of fit of the logistic regression model. Using census data of the regional population by gender, age, and education we computed a weight for each characteristic, comparing the population and the sample distribution. We weighted the sample data and generated the frequency distribution after the data were weighted [21]. The same approach was followed to analyse data for depression, anxiety, and physical symptoms.

Where data were missing, in prevalence analyses of evaluated characteristics, we used pairwise deletion, so that all variable data were used, and in analyses of OR, we used listwise deletion, so that data from the same participants were used in bivariate and multivariate models enabling comparison. Sensitivity analysis was performed by running two separate models, adding confounders with missing values. Statistical significance was evaluated using 95% confidence interval and a two-tailed *p*-value of < 0.05.

### Spatial analysis

A list of factors characterized the Lombardy region as an outlier in Italy, and worldwide, in the COVID-19 pandemic (e.g. number of cases and deaths) [22]. Considering this fact, and the fact that research has suggested that the impact of a crisis spreads out in a circle and declines gradually over geographical distance, a phenomenon known as the ripple effect, we analysed the relationship between the distance from the city of residence of the responders to the red zone in Lombardy, and their CPDI score [23–25].

For the residents of the Lombardy Region we calculated the distance between their municipality and the epicentre of the red zone of the two nearby towns of Alzano Lombardo and Nembro.

We evaluated the relationship between the result of the CDPI test (mild to severe vs normal) and the distance from the epicentre. To do this, we classified as “exposed” the residents within a threshold distance, and as “non-exposed” the residents outside the threshold distance. We fixed the initial threshold at a distance of 5 km from the epicentre and then incremented the threshold by 5 km at a time.

We evaluated the ORs, first considering the pathologic vs. normal result, then also considering mild/moderate vs. normal, and severe vs. normal. We evaluated a logistic regression model including the distance band coded in these 3 levels, to evaluate the determinants of the positive result of the test. We also produced a map of the Lombardy Region that showed the distance bands from the epicentre and that reported a dot for every survey reply placed on a random point within the municipality of residence, with a different color based on the 3 possible test results: normal, mild/moderate, or severe.

Geographic information system (GIS) software was used to generate maps (ArcGIS Desktop 10.3.1; Esri, Redlands, CA) that illustrate the geographic distribution in Lombardy of the residential area (municipality) of participants during the quarantine, the location of the COVID-19 positive cases, and the red zone by centroid geospatial resource.

We used SAS software, version 9.4 (SAS, Institute Inc., Cary, NC, USA) for all statistical analyses, accounting for stratification, clustering, and weighting of the study dataset.

### Ethics

This study was designed as descriptive epidemiological research. All data were encrypted. Formal ethical review board approval was not required for the present analysis of the data. The present research, however, was approved by the Institutional Review Board of the Istituto di Ricerche Farmacologiche Mario Negri IRCCS in Milan, Italy.

All the items of the STROBE checklist for cross-sectional studies have been met in the present report.

## Results

In total, 20,518 responders completed the online survey, and, of these, 30.1% were male and 69.9% were female. A total of 69.9% of the responders were 18–50 years old, 54.3% had a tertiary level of educational qualification, 69.5% were workers, 84.1% were living during the quarantine in houses with 3 or more rooms, and 13.7% were living alone. A minority of responders (9.7%) had been in contact with COVID-19 positive people (%). The majority of the population, on the other hand, had not had new health problems during the previous 14 days (90.8%), and had left the house to run an errand in the previous week (63.9%) (Table 1).

**Table 1:** Characteristics of total responders by psychological distress score range

	Normal	Mild-Moderate	Severe	Total	OR^**a**^	CI 95%	p
**Gender**							
Male	3962 (38.7)	1895 (21.9)	150 (14.3)	6007 (30.1)	*Reference*		
Female	6277 (61.3)	6764 (78.1)	901 (85.7)	13,942 (69.9)	2.37	2.22–2.52	<.0001
*Missing*	*301*	*238*	*30*	*569*			
**Age**							
18–30	1500 (14.5)	1393 (16.1)	206 (19.7)	3099 (15.4)	1.03	0.95–1.12	0.4785
31–50	3993 (38.6)	3649 (42.1)	482 (46.2)	8124 (40.5)	*Reference*		
51–65	3577 (34.6)	2840 (32.7)	299 (28.6)	6716 (33.5)	0.85	0.80–0.91	<.0001
≥66	1277 (12.3)	793 (9.1)	57 (5.5)	2127 (10.6)	0.64	0.58–0.71	<.0001
*Missing*	*193*	*222*	*37*	*452*			
**Education**							
First level	591 (5.8)	561 (6.5)	87 (8.3)	1239 (6.2)	1.25	1.11–1.40	0.0003
Second level	3866 (38.0)	3493 (40.6)	464 (44.4)	7823 (39.5)	1.16	1.10–1.23	<.0001
University	5724 (56.2)	4545 (52.9)	495 (47.3)	10,764 (54.3)	*Reference*		
*Missing*	*359*	*298*	*35*	*692*			
**Work**							
Workers	7299 (70.1)	6080 (69.3)	698 (65.1)	14,077 (69.5)	*Reference*		
Students	689 (6.6)	704 (8.0)	120 (11.2)	1513 (7.5)	1.29	1.16–1.43	<.0001
Unemployed	330 (3.2)	420 (4.8)	81 (7.5)	831 (4.1)	1.64	1.42–1.89	<.0001
Others	2093 (20.1)	1565 (17.8)	174 (16.2)	3832 (18.9)	0.90	0.83–0.97	0.0024
*Missing*	*129*	*128*	*8*	*265*			
**House**							
1–2 rooms	1480(14.5)	1460 (16.9)	217 (20.8)	3157 (15.9)	1.23	1.14–1.33	<.0001
≥3 rooms	8731 (85.5)	7201 (83.1)	824 (79.2)	16,756 (84.1)	*Reference*		
*Missing*	*329*	*236*	*40*	*605*			
**Live with**							
Alone	1431 (13.8)	1191 (13.7)	147 (13.8)	2769 (13.7)	*Reference*		
2 components	3029 (29.2)	2334 (26.8)	245 (23.0)	5608 (27.8)	0.91	0.83–1.00	0.0447
3 components	2497 (24.1)	2187 (25.1)	244 (22.9)	4928 (24.5)	1.04	0.95–1.14	0.3950
More than 3 components	3410 (32.9)	3003 (34.5)	428 (40.2)	6841 (34.0)	1.08	0.99–1.18	0.3950
*Missing*	*173*	*182*	*17*	*372*			
**New health problems during previous 14 days**							
Nothing	9528 (93.4)	7638 (88.8)	863 (82.3)	18,029 (90.8)	*Reference*		
Symptomatics	480 (4.7)	748 (8.7)	155 (14.8)	1383 (7.0)	2.11	1.88–2.36	<.0001
Non symptomatics	192 (1.9)	218 (2.5)	30 (2.9)	440 (2.2)	1.45	1.20–1.75	0.0001
*Missing*	*340*	*293*	*33*	*666*			
**Contact with Covid-19 positive people**							
Yes	861 (8.3)	943 (10.8)	151 (14.2)	1955 (9.7)	1.38	1.26–1.52	<.0001
No	9515 (91.7	7828 (89.2)	915 (85.8)	18,258 (90.3)	*Reference*		
*Missing*	*164*	*126*	*15*	*305*			
**Left the house in the previous week**							
Yes	6852 (67.1)	5285 (61.6)	546 (52.1)	12,683 (63.9)	*Reference*		
No	3362 (32.9)	3293 (38.4)	501 (47.9)	7156 (36.1)	1.33	1.25–1.41	<.0001
*Missing*	*326*	*319*	*34*	*679*			
**Total**	**10,540 (51.4)**	**8897 (43.4)**	**1081 (5.3)**	**20,518 (100)**			

^a^*Parameter Reference:* Mild-Moderate/Severe vs Normal

Of all responders, 9978 (48.6%) reported a psychological impact of any degree**.** More specifically, 8897 (43.4%) reported mild or moderate psychological impact and 1081 (5.2%) reported severe impact. In the univariate analysis, all the considered variables were statistically significant associated with psychological distress (Table 1).

The multivariate analysis after adjustments showed that an increasing CPDI score was associated, with a more than 2 fold value, with females (OR = 2.20; 95% CI 2.05–2.36), with people with a first (OR = 1.25; 95% CI 1.09–1.43) and second educational level (OR = 1.24; 95% CI 1.12–1.37), with unemployed people (OR = 1.32; 95% CI 1.14–1.53), with people living in a ≤2 room house (OR = 0.93; 95% CI 0.88–0.97), with having had new health problems during the previous14 days (OR = 0.93; 95% CI 0.88–0.97), and with not having been out of the house in the previous week (OR = 1.48; 95% CI 1.38–1.60) (Table 2). Sensitivity analysis where dependent variables with numbers of missing values of all considered variables were added did not change the overall results of the study (Table 2).

**Table 2:** Results of the logistic regression model for total responders by psychological distress

	Model 1			Model 2			Model 3		
	OR	CI 95%	p	OR	CI 95%	p	OR Weighted for residence, gender, age and education	CI 95%	p
**Gender**									
Female vs Male	*2.31*	*2.15–2.48*	*<.0001*	*2.35*	*2.20–2.51*	*<.0001*	*2.20*	*2.05–2.36*	*<.0001*
Missing vs Male				*1.66*	*1.39–1.98*	*<.0001*			
**Age**									
18–30 vs 31–50	*1.06*	*0.95–1.19*	*0.3183*	*1.01*	*0.92–1.12*	*0.7863*	*0.95*	*0.82–1.09*	*0.4577*
51–65 vs 31–50	*0.87*	*0.81–0.94*	*0.0006*	*0.86*	*0.80–0.92*	*<.0001*	*0.94*	*0.86–1.03*	*0.1687*
≥66 vs 31–50	*0.79*	*0.68–0.92*	*0.0020*	*0.77*	*0.68–0.88*	*<.0001*	*0.95*	*0.83–1.10*	*0.4967*
Missing vs 31–50				*1.04*	*0.84–1.27*	*0.7448*			
**Education**									
First level vs University	*1.30*	*1.13–1.50*	*0.0003*	*1.39*	*1.23–1.58*	*<.0001*	*1.25*	*1.09–1.43*	*0.0011*
Second level vs University	*1.23*	*1.14–1.31*	*<.0001*	*1.22*	*1.15–1.30*	*<.0001*	*1.24*	*1.12–1.37*	*<.0001*
Missing vs University				*1.09*	*0.93–1.29*	*0.2907*			
**Work**									
Student vs Workers	*1.09*	*0.93–1.27*	*0.2984*	*1.16*	*1.01–1.32*	*0.0348*	*0.95*	*0.80–1.13*	*0.5555*
Unemployed vs Workers	*1.49*	*1.26–1.76*	*<.0001*	*1.50*	*1.29–1.73*	*<.0001*	*1.32*	*1.14–1.53*	*0.0002*
Other vs Workers	*0.97*	*0.87–1.08*	*0.6071*	*0.96*	*0.87–1.06*	*0.3817*	*0.92*	*0.82–1.03*	*0.1414*
Missing vs Workers				*1.10*	*0.86–1.42*	*0.4519*			
**House**									
1–2 Rooms vs ≥3 Rooms	*1.13*	*1.03–1.25*	*0.0115*	*1.17*	*1.07–1.27*	*0.0004*	*1.45*	*1.31–1.60*	*<.0001*
Missing vs ≥3 Rooms				*0.91*	*0.77–1.09*	*0.3149*			
**Live with**									
2 components vs Alone	*0.89*	*0.80–1.00*	*0.0416*	*0.93*	*0.85–1.03*	*0.1616*	*0.85*	*0.76–0.96*	*0.0061*
3 components vs Alone	*1.02*	*0.91–1.14*	*0.7439*	*1.02*	*0.92–1.13*	*0.6565*	*1.08*	*0.96–1.22*	*0.1953*
More than 3 components vs Alone	*1.00*	*0.90–1.12*	*0.9601*	*1.02*	*0.92–1.12*	*0.7722*	*1.08*	*0.96–1.22*	*0.1948*
Missing vs Alone				*1.27*	*1.01–1.60*	*0.0404*			
**New health problems during previous 14 days**									
Symptomatic vs Nothing	*2.00*	*1.75–2.29*	*<.0001*	*1.97*	*1.75–2.23*	*<.0001*	*2.01*	*1.65–2.44*	*<.0001*
Non symptomatic vs Nothing	*1.32*	*1.05–1.66*	*0.0181*	*1.29*	*1.05–1.58*	*0.0141*	*2.40*	*1.80–3.20*	*<.0001*
Missing vs Nothing				*0.99*	*0.84–1.17*	*0.9399*			
**Contact with Covid-19 positive people**									
Yes vs No	*1.20*	*1.07–1.34*	*0.0022*	*1.20*	*1.09–1.33*	*0.0004*	*1.09*	*0.92–1.31*	*0.3202*
Missing vs No				*0.91*	*0.72–1.15*	*0.4209*			
**Left the house in the previous week**									
No vs Yes	*1.17*	*1.09–1.25*	*<.0001*	*1.18*	*1.11–1.25*	*<.0001*	*1.48*	*1.38–1.60*	*<.0001*
Missing vs Yes				*1.22*	*1.04–1.43*	*0.0150*			

Hosmer and Lemeshow Goodness of Fit test:

Model 1: χ^2^= 3.4632; df =8; *p-*value=0.9020

Model 2: χ^2^= 9.7469; df =8; *p*-value=0.2832

Model 3: χ^2^=78.7974; df =8; *p-*value=< 0.0001

Concerning the type of psychological distress, 2003 responders (9.9%) reported moderate to severe depressive symptoms, 1131 (5.5%) reported moderate to severe anxiety symptoms, and 802 (3.9%) reported moderate to severe physical symptoms. Following the same approach, and applying it to the analysis of all levels of psychological distress reported above, the multivariate analyses after adjustments showed that increasing CPDI score for depressive, anxiety, and physical symptoms was statistically significant when associated with gender, education, work, housing, new health problems during the previous 2 weeks, and not having been out of the house in the previous week (Additional Tables 2-7).

**Fig. 1:**
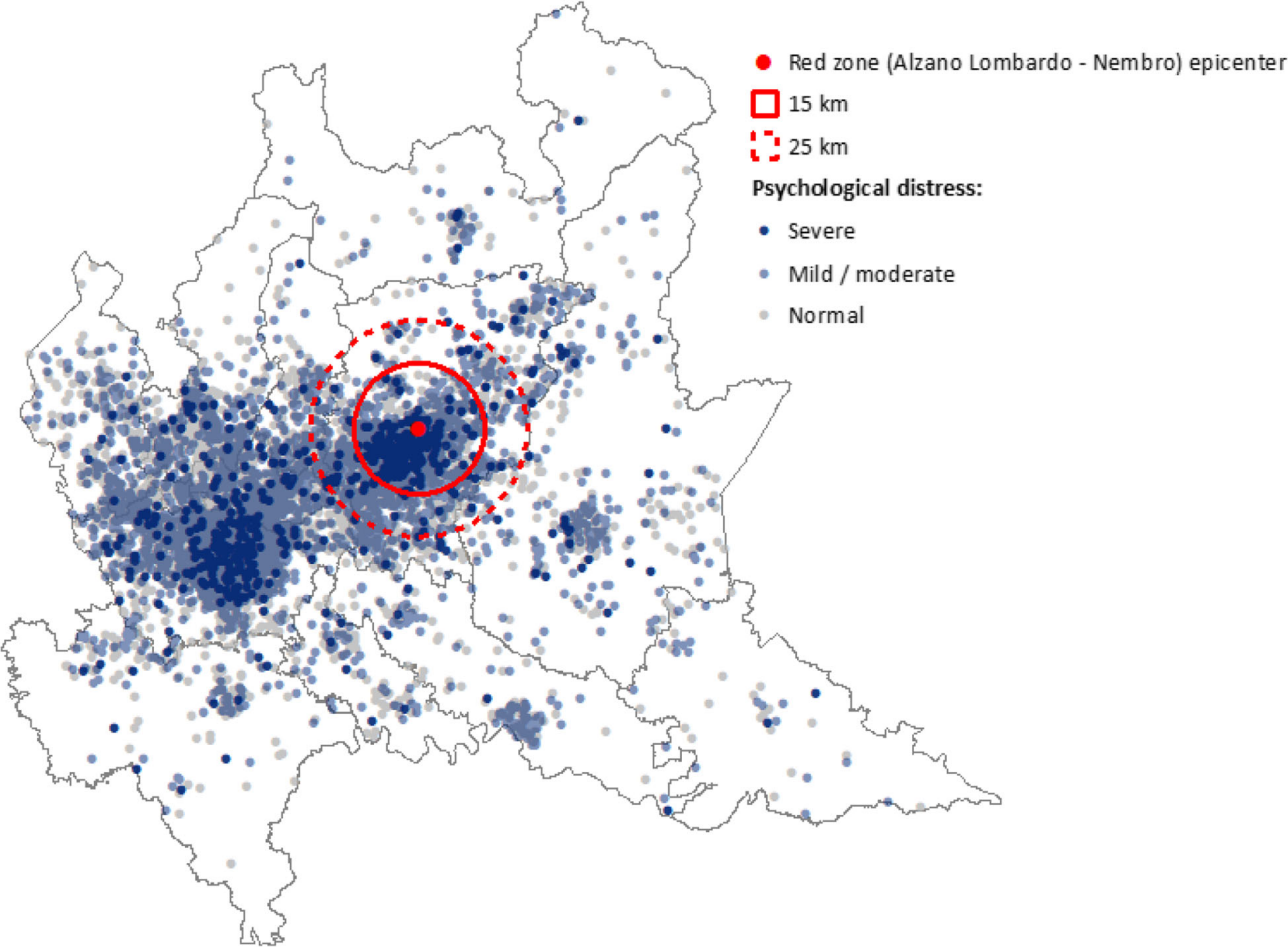
Distribution of responders in the Lombardy Region according to the grade of psychological distress

A positive correlation was found between responder rate (per 10.000 residents) and positive COVID-19 cases (per 10.000 residents) by region (r_s_ = + 0.83, *p =* < 0.0001). A positive correlation was also found between responder rate and region latitude (r_s_ = + 0.91, *p =* < 0.0001), with a greater response rate in the north of Italy. Taking into account the data from the 14,597 responders from the Lombardy Region (the most represented and representative in the survey, and the one that suffered the most from the virus in Italy and in the world at the time of the survey), a focused analysis was performed on the distance from the residence of the responder population to the red zone. The median distance was 45.3 km. Responders in or next to the red zone in the Lombardy Region reported a higher prevalence of psychological distress than those in other zones of the region (54.3 and 48.5%, respectively; OR = 1.23, 95% CI 1.05–1.43) (Fig. 1). Distance was associated with psychological distress and with its extent: increased distance of the place of residence to the red zone decreased the likelihood of psychological distress in the population, both for mild/moderate and severe distress. For up to 15 km of distance the OR = 1.40 (95% CI 1.29–1.53) and from 15 to 25 km the OR = 1.28 (95% CI 1.12–1.47), compared to the > 25 km situation (Table 3).

**Table 3:** Distance from the red zone in the Lombardy Region and psychological distress in the population

Psychological distress							
Distance	Normal	Mild-Moderate	Severe	Total	OR^**a**^	CI 95%	***p***-value
≤ 15 km	1293 (22.5)	1338 (27.6)	201 (19.1)	2832 (25.3)	1.40	1.29–1.53	*<.0001*
16–25 km	444 (7.7)	427 (8.8)	55 (49.5)	926 (8.4)	1.28	1.12–1.47	*0.0004*
≥ 26 km	4015 (69.8)	3081 (63.6)	163 (19.6)	7420 (66.3)	*Reference*		
*Missing*	*1921*	*1250*	*409*	*3419*			
**Total**	**7673 (52.6)**	**6096 (41.7)**	**828 (5.7)**	**14,597 (100)**			

^a^*Parameter Reference:* Mild-Moderate/Severe vs Normal

## Discussion

This study is among the first sample studies to track psychological changes in the Italian population during the COVID-19 quarantine. It is natural for everyone to feel fear, sadness, and anxiety during a crisis. The COVID-19 pandemic affects the population in many ways and not only threatens physical health, it also affects mental health [26, 27]. Overall, our results support previous research that followed prior pandemics. The increased need for psychological and psychiatric care suggests that appropriate mental health services should be alerted and organized to support people during and after quarantine [28].

Concerning influential factors on psychological distress during quarantine, females experienced more distress also in the present study [11, 29, 30]. Research data often report higher levels of psychological distress in women in the general population. Gender is an important biological determinant of vulnerability to psychosocial stressors: women are more vulnerable to distress and more likely to develop post-stress symptoms over time [31]. In particular, a recent review examined economic, occupational, and familial stressors that result in gender-based disparities during the COVID-19 pandemic [32]. Since the pandemic began, women have experienced job loss and underemployment with greater propensity. Women also comprise the majority of healthcare workers within the U.S. and thus are also at heightened risk for occupational exposure. Additionally, due to school closures during the pandemic, women have disproportionately experienced caregiver burden. Both increased responsibilities at work and at home as a result of the pandemic, and the application of a range of coping mechanisms, such as disengagement, denial, and energy conservation, have been described [33]. This study highlighted the increased psychological manifestation of stress and burnout for women in response to a significantly enhanced “second shift” because of the pandemic and lockdown context. Moreover, the gender demographic bias in our study may, in this context, have led to an elevation in the levels of psychological distress reported in this study. The high proportion of women in the sample, as in similar previous studies [30, 34, 35], may be due to greater interest and participation in studies of this nature. An adverse psychological impact was felt in quarantine both in males and females, however [7].

Studies have reported participant fear about their own health or fear of infecting others, and have reported that participants in quarantine were more likely to fear infecting family members than those not quarantined [36]. Living alone or with other people, as with living in houses with ≤2 rooms compared to more rooms, can be potential risk factors for the onset of psychological distress symptoms as reported in the present study.

Confinement, loss of usual routine, and reduced social and physical contact with others were frequently shown to cause boredom, frustration, and a sense of isolation from the rest of the world, which was distressing to participants. This frustration was exacerbated by not being able to take part in usual day-to-day activities, such as shopping for basic necessities or taking part in social networking activities via the telephone or internet [37]. According to other research, quarantine had an impact on keeping track of time, led to confusion in people confined in their houses, and created sense of boredom and the feeling of being stuck in time [38]. The reduced incidence of psychological distress observed in the present study in people who left the house during quarantine supports this.

People who were isolated for 2 weeks due to contacts with COVID-19 suffered from high rates of anxiety symptoms and anger during isolation, and showed mental health effects [39]. People quarantined for more than 10 days showed significantly higher post-traumatic stress symptoms than those quarantined for less than 10 days [40].

In the present study 48.6% of responders reported psychological distress, a middle rate between the 11.5% reported in the Nepalese population and the 71,3 in Brazilians [17, 18]. 43.4% of responders were mildly/moderately distressed, and 5.2% severely distressed. Also the rates of distress of different levels varied between the countries that used the same self-reported questionnaire (CPDI). This difference may reflect a different impact of the COVID-19 pandemic between countries (i.e. death rate), but may also reflect differences in countries’ medical systems, availability of personal protective equipment, timeliness and intensity of interventions, lockdown policies, and spread of information through mainstream and social media.

Overall, the rate of responders experiencing psychological distress was about double that observed in the general Italian non-quarantined population, also for the investigated type of distress [41]. The same results emerged in other Italian surveys conducted in the same period: the majority of respondents reported from moderate to high level of depression, general anxiety, and stress symptomatology associated with long home confinement [13, 29, 35, 42].

The positive correlation between responder rate and region latitude, with a greater response rate in the north, may reflect the exposure and involvement of those people living in zones where the virus hit harder. According to another Italian survey, people living in most affected region showed more psychological distress, anxiety, and depression symptoms compare to southern Italian respondents [35]. The perception of the presence of the virus, such as the daily exposure to bad news about death and increasing positive cases nearby, may have caught more attention on this topic and promoted survey attendance. The effects of quarantine were different in those living far away from the epicentre of the crisis. In fact, a higher prevalence of psychological distress was found up to 25 km away from the red zone and, in particular, severe distress was found within a 15 km range.

The findings of the present study are in agreement with recent rapid reviews [7, 8, 27, 43] that reported a high burden of mental health symptoms linked to the COVID-19 pandemic and quarantine condition. Addressing mental health in public health emergencies is therefore of vital importance. Past experience does not appear to have been effective in facing the COVID-19 pandemic. Preparedness before the next emergency is essential, and not only for mental health.

### Strengths and limitations

This study is one of the few surveys on psychological distress during the COVID-19 pandemic, and has the broadest participation level and largest sample size. The observed relationship between responder rate (per 10.000 residents) and positive COVID-19 cases (per 10.000 residents) by region at mid-survey is an additional strength of the study, supporting the idea that the study succeeded in intercepting the bearers of needs.

A web-based format was chosen over random-digit dialling for both cost considerations and time constraints. Research to date has shown that Internet-mediated questionnaires have reliability and internal validity characteristics that are proportionate to traditional formats [44, 45]. We adopted the snowball sampling strategy, with its strengths and weaknesses. Consequently, it was not possible to assess the participation rate since the number of subjects who received the link to the survey wa unknown. The project was initiated and completed soon after the start of the quarantine, without a funding source, at a time when concerns about COVID-19 were still a part of daily life in Italy. Online data collection has considerable potential, but the large sample of participants may not be representative of the Italian general population by residence, age, sex, education, and a variety of other characteristics. Most of the participants (58% of completed questionnaires) were from the Lombardy Region, the most affected. To consider this bias we weighted data for gender, age, and education of the national population by region to achieve a representative sample [34], and findings from raw or weighted data analyses were similar. Characteristics of responders may be correlated with their exposure and perceptions of COVID-19 as well with as their decision to participate in the study. An additional response bias may therefore exist if the non-responders were either too stressed to respond or not at all stressed, and therefore not interested in the survey. In Italy, an average of 67,2% of the people used the internet in 2019, ranging from 75% of inhabitants in the Trentino-Alto Adige to 62.1% in the Calabria regions, following a North to South trend [46]. Online research thus misses part of the overall population. High-functioning users of social media who are already engaged in similar initiatives might be over-represented. At the same time, the present study is representative of the adult internet population. Lastly, the use of cross-sectional self-reported data, as in the present study, precludes attribution of causality. This was not the aim of the present study, however. The findings reflect important associations among the variables we studied, and strong corroboration between these findings and existing literature about previous quarantines [25, 47–50], suggesting the need for future longitudinal studies in this area, also to verify whether psychological distress might occur with COVID-19 progression and sequences [26, 51–57].

## Conclusions

Despite some methodological limitations, the consistency and direction of the statistical results, as well as their agreement with results of similar surveys conducted in different countries, support the fact that this study was able to determine the psychological impact of quarantine in Italy for the COVID-19 pandemic. Study findings indicated that the quarantine affected the population, producing psychological distress in close to half of responders. Statistical analyses indicated that certain groups were more vulnerable to psychological distress: females, young adults, those with no formal educational qualifications, the unemployed, those living alone, and those with new, recent health problems.

Overall, the study suggests that the psychological impact of quarantine is substantial. Depriving people of their liberty for the wider public good needs to be handled carefully. If quarantine is essential, the present findings suggest that people need to understand what is happening, why, and for how long. A uniform quarantine law applies to the whole country, but Italy is characterized by different jurisdictions, self-government of health services, and different COVID-19 prevalence rates between and within regions. This heterogeneity can produce different levels of acceptability, and different results at the local level. An epidemic is capable of involving regions with multiple jurisdictions in common, shared initiatives to improve health system organization and governance. The regional leaders will need to coordinate their quarantine measures and messages to ensure consistency. Variations in these measures and different interpretations of the messages provided will produce confusion among residents and damage the legitimacy of the quarantine, even among those who want to comply. Clear communication should be provided on how to face the period of restrictions, and should reinforce the sense of altruism that everyone should be feeling. Individuals will not be in the same situations so health officials and policy makers have the responsibility to work towards an appropriate quarantine management also to prevent adverse health (also psychological) and social outcomes on the short and long term.

Public health officials, infectious diseases physicians, psychologists, and psychiatrists must work on the adverse psychological and social consequences of quarantine, and must do so now, to reduce, and, in the future, to prevent them [37, 40, 47, 58].

The COVID-19 pandemic has brought up numerous, interesting research questions that should be addressed in the future. Concerning mental health care, for example, the pandemic has seen a massive increase in the use of telepsychiatry. It would be useful to evaluate the quality and efficacy of this intervention. The outcomes of telepsychiatry in real-life settings should be evaluated and compared to regular face-to-face therapy [59]. Research is also needed to assess and monitor the implications of enacted policies. Priorities and longer-term strategies for mental health science research with a multidisciplinary approach are imperative [28]. The mental health effects of COVID-19 on the general population and people living with mental illness deserve serious attention and are needs that should be prioritised. Interventions should be community-based with a multidisciplinary and multisectoral approach as part of a social, collective effort to tackle the social problems caused by COVID-19 [60].

Countries vary widely in terms of their capacity to prevent, detect, and respond to outbreaks [61]. The comparison of outbreak characteristics between countries is influenced by potential confounders such as different phases of outbreaks, mean age of the affected population, management of the pandemic, amount of tests administered, definitions of COVID-19–related deaths, and underreporting. The COVID-19 outbreak, however, is once again an opportunity for each affected country to better set up its preparedness for future health emergencies, also foreseeing appropriate initiatives to prevent psychological distress in the population.

Unfortunately, lessons of previous coronavirus epidemics (severe acute respiratory syndrome, SARS; Middle East respiratory syndrome; MERS) concerning the epidemics’ association with a psychiatric burden in both the acute and post-illness stages [39], seem not to have been learnt. In conclusion, we must be certain that community and personalized interventions to improve resilience and reduce the risk of psychopathology in the current crisis are promptly implemented. A prompt recovery after the crisis is desirable, as well as an effective preparedness before the next emergency.

## Supplementary Information

**Additional file 1: Table S1.** COVID-19 Peritraumatic Distress Index (CPDI) questionnaire. **Table s2.** Characteristics of total responders by depression. **Table S3.** Results of the logistic regression model for total responders (depression). **Table s4.** Characteristics of total responders by anxiety. **Table S5.** Results of logistic regression model for total responders (anxiety). **Table S6.** Characteristics of total responders by physical symptoms. **Table S7.** Results of the logistic regression model for total responders (physical symptoms).

## Data Availability

The datasets analysed during the current study are available from the corresponding author on reasonable request.

## Abbreviations

COVID-19: Coronavirus disease 2019
CPDI: COVID-19 Peritraumatic Distress Index
CMS: Content management system
OR: Odds ratios
CI: Confidence intervals
GIS: Geographic information system
SD: Standard deviation
PTSD: Post-traumatic stress disorder
SARS: Severe acute respiratory syndrome
MERS: Middle East respiratory syndrome
