# Supplementary material for: Psychological distress among Italians during the 2019 coronavirus disease (COVID-19) quarantine
Source: BMC Psychiatry. 2021 Jan 8;21:20. doi: 10.1186/s12888-020-03027-8 (PMC7793386; doi:10.1186/s12888-020-03027-8)
Supplement: Supplementary file 1 — Additional file 1: Table S1. COVID-19 Peritraumatic Distress Index (CPDI) questionnaire. Table s2. Characteristics of total responders by depression. Table S3. Results of the logistic regression model for total responders (depression). Table s4. Characteristics of total responders by anxiety. Table S5. Results of logistic regression model for total responders (anxiety). Table S6. Characteristics of total responders by physical symptoms. Table S7. Results of the logistic regression model for total responders (physical symptoms). [file 12888_2020_3027_MOESM1_ESM.docx]

**Additional Table 1.** COVID-19 Peritraumatic Distress Index (CPDI) questionnaire

| **ID** | **Question** | **Answer** | **value** |
| --- | --- | --- | --- |
| S1 |  | never | 0 |
|  |  | occasionally | 1 |
|  | Compared to usual, I feel more nervous and anxious | sometimes | 2 |
|  |  | often | 3 |
|  |  | most of the time | 4 |
| S2 | I feel insecure and bought a lot of masks, medications, sanitizer, gloves and/or other home supplies | never | 0 |
|  |  | occasionally | 1 |
|  |  | sometimes | 2 |
|  |  | often | 3 |
|  |  | most of the time | 4 |
| S3 | I can’t stop myself from imagining myself or my family being infected and feel terrified and anxious about it | never | 0 |
|  |  | occasionally | 1 |
|  |  | sometimes | 2 |
|  |  | often | 3 |
|  |  | most of the time | 4 |
| S4 |  | never | 0 |
|  |  | occasionally | 1 |
|  | I feel empty and helpless no matter what I do | sometimes | 2 |
|  |  | often | 3 |
|  |  | most of the time | 4 |
| S5 |  | never | 0 |
|  | I feel sympathetic to the COVID-19 patients and their families. I feel sad about them. | occasionally | 1 |
|  |  | sometimes | 2 |
|  |  | often | 3 |
|  |  | most of the time | 4 |
| S6 |  | never | 0 |
|  |  | occasionally | 1 |
|  | I feel helpless and angry about people around me, governors, and media | sometimes | 2 |
|  |  | often | 3 |
|  |  | most of the time | 4 |
| S7 |  | never | 0 |
|  | I am losing faith in the people around me | occasionally | 1 |
|  |  | sometimes | 2 |
|  |  | often | 3 |
|  |  | most of the time | 4 |
| S8 | I collect information about COVID-19 all day. Even if it’s not necessary, I can’t stop myself | never | 0 |
|  |  | occasionally | 1 |
|  |  | sometimes | 2 |
|  |  | often | 3 |
|  |  | most of the time | 4 |
| S9 | I believe the COVID-19 information from any source without any evaluation | never | 0 |
|  |  | occasionally | 1 |
|  |  | sometimes | 2 |
|  |  | often | 3 |
|  |  | most of the time | 4 |
| S10 | I would rather believe in negative news about COVID-19 and be sceptical about the good news | never | 0 |
|  |  | occasionally | 1 |
|  |  | sometimes | 2 |
|  |  | often | 3 |
|  |  | most of the time | 4 |
| S11 |  | never | 0 |
|  |  | occasionally | 1 |
|  | I am constantly sharing news about COVID-19 (mostly negative news) | sometimes | 2 |
|  |  | often | 3 |
|  |  | most of the time | 4 |
| S12 |  | never | 0 |
|  |  | occasionally | 1 |
|  | I avoid watching COVID-19 news, since I am too scared to do so | sometimes | 2 |
|  |  | often | 3 |
|  |  | most of the time | 4 |
| S13 |  | never | 0 |
|  |  | occasionally | 1 |
|  | I am more irritable and have frequent conflicts with my family | sometimes | 2 |
|  |  | often | 3 |
|  |  | most of the time | 4 |
| S14 |  | never | 0 |
|  |  | occasionally | 1 |
|  | I feel tired and sometimes even exhausted | sometimes | 2 |
|  |  | often | 3 |
|  |  | most of the time | 4 |
| S15 |  | never | 0 |
|  |  | occasionally | 1 |
|  | Due to feelings of anxiety, my reactions are becoming sluggish. | sometimes | 2 |
|  |  | often | 3 |
|  |  | most of the time | 4 |
| S16 |  | never | 0 |
|  |  | occasionally | 1 |
|  | I find it hard to concentrate | sometimes | 2 |
|  |  | often | 3 |
|  |  | most of the time | 4 |
| S17 |  | never | 0 |
|  |  | occasionally | 1 |
|  | I find it hard to make any decisions | sometimes | 2 |
|  |  | often | 3 |
|  |  | most of the time | 4 |
| S18 |  | never | 0 |
|  |  | occasionally | 1 |
|  | During this COVID-19 period, I often feel dizzy or have back pain and chest distress | sometimes | 2 |
|  |  | often | 3 |
|  |  | most of the time | 4 |
| S19 |  | never | 0 |
|  | During this COVID-19 period, I often feel stomach pain, bloating, and other stomach discomfort | occasionally | 1 |
|  |  | sometimes | 2 |
|  |  | often | 3 |
|  |  | most of the time | 4 |
| S20 |  | never | 0 |
|  |  | occasionally | 1 |
|  | I feel uncomfortable when communicating with others | sometimes | 2 |
|  |  | often | 3 |
|  |  | most of the time | 4 |
| S21 |  | never | 0 |
|  |  | occasionally | 1 |
|  | Recently, I rarely talk to my family | sometimes | 2 |
|  |  | often | 3 |
|  |  | most of the time | 4 |
| S22 |  | never | 0 |
|  | I cannot sleep well. I always dream about myself or my family being infected by COVID-19 | occasionally | 1 |
|  |  | sometimes | 2 |
|  |  | often | 3 |
|  |  | most of the time | 4 |
| S23 |  | never | 0 |
|  |  | occasionally | 1 |
|  | I lost my appetite | sometimes | 2 |
|  |  | often | 3 |
|  |  | most of the time | 4 |
| S24 |  | never | 0 |
|  |  | occasionally | 1 |
|  | I have constipation or frequent urination | sometimes | 2 |
|  |  | often | 3 |
|  |  | most of the time | 4 |

|  | Raw Score | = sum of value of each question |
| --- | --- | --- |
| Introduction to: | Display Score | = Raw Score + 4 |
| Don’t panic about this coronavirus, keep calm. Let’s pay attention to our mental health by filling out this CPDI assessment.  Instructions: Please select the frequency of the below activities in the last week. Now, let’s start! | Cutoff Score  (Display Score) | Normal: 0-28 Mild: 29-52 Severe: 53-100 |

**Additional Table 2.** Characteristics of total responders by depression

|  | **Yes** | | **No** | | **Total** | | **OR** | | **CI 95%** | | **p-value** | |  |
| --- | --- | --- | --- | --- | --- | --- | --- | --- | --- | --- | --- | --- | --- |
| **Gender** |  | |  | |  | |  | |  | |  | |  |
| Male | 1611 (83.0) | | 12331 (68.5) | | 13942 (69.9) | | *Reference* | |  | |  | |  |
| Female | 329 (17.0) | | 5678 (31.5) | | 6007 (30.1) | | *2.26* | | *1.99-2.55* | | *<.0001* | |  |
| *Missing* | *63* | | *506* | | *569* | |  | |  | |  | |  |
| **Age** |  | |  | |  | |  | |  | |  | |  |
| 18-30 | 509 (26.5) | | 2590 (14.3) | | 3099 (15.4) | | *1.67* | | *1.48-1.88* | | *<.0001* | |  |
| 31-50 | 855 (44.6) | | 7269 (40.1) | | 8124 (40.5) | | *Reference* | |  | |  | |  |
| 51-65 | 470 (24.5) | | 6246 (34.4) | | 6716 (33.5) | | *0.64* | | *0.57-0.72* | | *<.0001* | |  |
| ≥66 | 85 (4.4) | | 2042 (11.3) | | 2127 (10.6) | | *0.35* | | *0.28-0.45* | | *<.0001* | |  |
| *Missing* | *84* | | *368* | | *452* | |  | |  | |  | |  |
| **Education** |  | |  | |  | |  | |  | |  | |  |
| First level | 115 (6.0) | | 1124 (6.3) | | 1239 (6.2) | | *0.98* | | *0.80-1.21* | | *0.8742* | |  |
| Second level | 802 (41.5) | | 7021 (39.2) | | 7823 (39.5) | | *1.10* | | *1.00-1.21* | | *0.0595* | |  |
| University | 1014 (52.5) | | 9750 (54.5) | | 10764 (54.3) | | *Reference* | |  | |  | |  |
| *Missing* | *72* | | *620* | | *692* | |  | |  | |  | |  |
| **Work** |  | |  | |  | |  | |  | |  | |  |
| Workers | 1292 (65.3) | | 12785 (70.0) | | 14077 (69.5) | | *Reference* | |  | |  | |  |
| Students | 298 (15.1) | | 1215 (6.6) | | 1513 (7.5) | | *2.43* | | *2.11-2.79* | | *<.0001* | |  |
| Unemployed | 138 (7.0) | | 693 (3.8) | | 831 (4.1) | | *1.97* | | *1.63-2.39* | | *<.0001* | |  |
| Others | 251 (12.7) | | 3581 (19.6) | | 3832 (18.9) | | *0.69* | | *0.60-0.80* | | *<.0001* | |  |
| *Missing* | *24* | | *241* | | *265* | |  | |  | |  | |  |
| **House** |  | |  | |  | |  | |  | |  | |  |
| 1-2 rooms | 379 (19.5) | | 2778 (15.5) | | 3157 (15.9) | | *1.33* | | *1.18-1.50* | | *<.0001* | |  |
| ≥3 rooms | 1562 (80.5) | | 15194 (84.5) | | 16756 (84.1) | | *Reference* | |  | |  | |  |
| *Missing* | *62* | | *543* | | *605* | |  | |  | |  | |  |
| **Live with** |  | |  | |  | |  | |  | |  | |  |
| Alone | 266 (13.5) | | 2503 (13.8) | | 2769 (13.7) | | *Reference* | |  | |  | |  |
| 2 components | 423 (21.5) | | 5185 (28.5) | | 5608 (27.8) | | *0.77* | | *0.65-0.90* | | *0.0013* | |  |
| 3 components | 463 (23.6) | | 4465 (24.6) | | 4928 (24.5) | | *0.98* | | *0.83-1.14* | | *0.7615* | |  |
| More than 3 components | 814 (41.4) | | 6027 (33.2) | | 6841 (34.0) | | *1.27* | | *1.10-1.47* | | *0.0013* | |  |
| *Missing* | *37* | | *335* | | *372* | |  | |  | |  | |  |
| **New health problems during previous 14 days** | |  | |  | |  | |  | |  | |  | |
| Nothing | | 1647 (85.1) | | 16382 (91.4) | | 18029 (90.8) | | *Reference* | |  | |  | |
| Symptomatics | | 235 (12.1) | | 1148 (6.4) | | 1383 (7.0) | | *2.04* | | *1.75-2.36* | | *<.0001* | |
| Non symptomatics | | 54 (2.8) | | 386 (2.2) | | 440 (2.2) | | *1.39* | | *1.04-1.86* | | *0.0252* | |
| *Missing* | | *67* | | *599* | | *666* | |  | |  | |  | |
| **Contact with Covid-19 positive people** | |  | |  | |  | |  | |  | |  | |
| Yes | | 257 (13.0) | | 1698 (9.3) | | 1955 (9.7) | | *1.45* | | *1.26-1.67* | | *<.0001* | |
| No | | 1721 (87.0) | | 16537 (90.7) | | 18258 (90.3) | | *Reference* | |  | |  | |
| *Missing* | | *25* | | *280* | | *305* | |  | |  | |  | |
| **Left the house in the previous week** | |  | |  | |  | |  | |  | |  | |
| Yes | | 1075 (55.1) | | 11608 (64.9) | | 12683 (63.9) | | *Reference* | |  | |  | |
| No | | 876 (44.9) | | 6280 (35.1) | | 7156 (36.1) | | *1.51* | | *1.37-1.66* | | *<.0001* | |
| *Missing* | | *52* | | *627* | | *679* | |  | |  | |  | |
| **Total** | | **2003 (9.8)** | | **18515 (90.2)** | | **20518 (100)** | |  | |  | |  | |

**Additional Table 3.** Results of the logistic regression model for total responders (depression)

|  | **Model 1** | | | **Model 2** | | | **Model 3** | | |
| --- | --- | --- | --- | --- | --- | --- | --- | --- | --- |
|  | **OR** | **CI 95%** | **p** | **OR** | **CI 95%** | **p** | **OR**  **Weighted**  **for residence, gender, age and education** | **CI 95%** | **p** |
| **Gender** |  |  |  |  |  |  |  |  |  |
| Female *vs* Male | 2.21 | 1.95-2.50 | <.0001 | 2.12 | 1.84-2.43 | <.0001 | 1.86 | 1.65-2.11 | <.0001 |
| Missing *vs* Male | 2.05 | 1.53-2.75 | <.0001 |  |  |  |  |  |  |
| **Age** |  |  |  |  |  |  |  |  |  |
| 18-30 *vs* 31-50 | 1.48 | 1.28-1.71 | <.0001 | 1.52 | 1.29-1.79 | <.0001 | 1.58 | 1.30-1.93 | <.0001 |
| 51-65 *vs* 31-50 | 0.67 | 0.59-0.76 | <.0001 | 0.64 | 0.55-0.73 | <.0001 | 0.55 | 0.46-0.65 | <.0001 |
| ≥66 *vs* 31-50 | 0.43 | 0.33-0.57 | <.0001 | 0.41 | 0.30-0.56 | <.0001 | 0.48 | 0.37-0.62 | <.0001 |
| Missing *vs* 31-50 | 1.50 | 1.14-1.97 | 0.0033 |  |  |  |  |  |  |
| **Education** |  |  |  |  |  |  |  |  |  |
| First level *vs* University | 1.28 | 1.04-1.59 | 0.0220 | 1.25 | 0.98-1.59 | 0.0763 | 1.22 | 0.95-1.56 | 0.1108 |
| Second level *vs* University | 1.17 | 1.05-1.30 | 0.0030 | 1.19 | 1.06-1.33 | 0.0031 | 1.20 | 1.01-1.43 | 0.0362 |
| Missing *vs* University | 1.16 | 0.89-1.51 | 0.2864 |  |  |  |  |  |  |
| **Work** |  |  |  |  |  |  |  |  |  |
| Student *vs* Workers | 1.49 | 1.24-1.78 | <.0001 | 1.41 | 1.14-1.74 | 0.0013 | 1.19 | 0.95-1.50 | 0.1261 |
| Unemployed *vs* Workers | 1.74 | 1.43-2.12 | <.0001 | 1.89 | 1.52-2.34 | <.0001 | 1.30 | 1.05-1.61 | 0.0175 |
| Other *vs* Workers | 1.03 | 0.87-1.22 | 0.7521 | 1.10 | 0.91-1.34 | 0.3232 | 1.27 | 1.04-1.54 | 0.0163 |
| Missing *vs* Workers | 0.96 | 0.63-1.49 | 0.8709 |  |  |  |  |  |  |
| **House** |  |  |  |  |  |  |  |  |  |
| 1-2 Rooms *vs* ≥3 Rooms | 1.20 | 1.05-1.37 | 0.0077 | 1.12 | 0.96-1.30 | 0.1574 | 0.92 | 0.78-1.09 | 0.3446 |
| Missing *vs* ≥3 Rooms | 1.12 | 0.84-1.49 | 0.4230 |  |  |  |  |  |  |
| **Live with** |  |  |  |  |  |  |  |  |  |
| 2 components *vs* Alone | 1.33 | 1.12-1.57 | 0.0008 | 1.34 | 1.11-1.62 | 0.0020 | 2.58 | 2.11-3.15 | <.0001 |
| 3 components *vs* Alone | 1.13 | 0.98-1.31 | 0.0850 | 1.14 | 0.97-1.34 | 0.1032 | 1.41 | 1.16-1.71 | 0.0005 |
| More than 3 components *vs* Alone | 1.36 | 1.19-1.55 | <.0001 | 1.31 | 1.13-1.52 | 0.0004 | 2.23 | 1.87-2.67 | <.0001 |
| Missing *vs* Alone | 1.20 | 0.83-1.74 | 0.3213 |  |  |  |  |  |  |
| **New health problems during previous 14 days** |  |  |  |  |  |  |  |  |  |
| Symptomatic *vs* Nothing | 1.80 | 1.53-2.11 | <.0001 | 1.76 | 1.47-2.10 | <.0001 | 1.37 | 1.04-1.79 | 0.0251 |
| Non symptomatic *vs* Nothing | 1.16 | 0.86-1.58 | 0.3321 | 1.25 | 0.89-1.76 | 0.1968 | 2.99 | 2.17-4.13 | <.0001 |
| Missing *vs* Nothing | 0.85 | 0.65-1.11 | 0.2396 |  |  |  |  |  |  |
| **Contact with Covid-19 positive people** |  |  |  |  |  |  |  |  |  |
| Yes *vs* No | 1.25 | 1.07-1.45 | 0.0045 | 1.30 | 1.10-1.54 | 0.0024 | 1.09 | 0.84-1.42 | 0.5061 |
| Missing *vs* No | 0.91 | 0.60-1.38 | 0.6458 |  |  |  |  |  |  |
| **Left the house in the previous week** |  |  |  |  |  |  |  |  |  |
| No *vs* Yes | 1.22 | 1.10-1.35 | <.0001 | 1.19 | 1.06-1.33 | 0.0023 | 1.62 | 1.44-1.83 | <.0001 |
| Missing *vs* Yes | 0.92 | 0.69-1.24 | 0.6042 |  |  |  |  |  |  |

Hosmer and Lemeshow Goodness of Fit test:

Model 1: χ^2^= 5.3313; df =8; p-value=0.7216

Model 2: χ^2^= 5.0445; df =8; p-value=0.7528

Model 3: χ^2^=65.1689; df =8; p-value=<0.0001

**Additional Table 4.** Characteristics of total responders by anxiety

|  | **Yes** | **No** | **Total** | **OR** | **CI 95%** | **p-value** |
| --- | --- | --- | --- | --- | --- | --- |
| **Gender** |  |  |  |  |  |  |
| Male | 925 (84.1) | 13017 (69.1) | 13942 (69.9) | *Reference* |  |  |
| Female | 175 (15.9) | 5832 (30.9) | 6007 (30.1) | *2.37* | *2.01-2.79* | *<.0001* |
| *Missing* | *31* | *538* | *569* |  |  |  |
| **Age** |  |  |  |  |  |  |
| 18-30 | 160 (14.5) | 2939 (15.5) | 3099 (15.4) | *0.88* | *0.73-1.06* | *0.1685* |
| 31-50 | 474 (42.9) | 7650 (40.3) | 8124 (40.5) | *Reference* |  |  |
| 51-65 | 379 (34.3) | 6337 (33.4) | 6716 (33.5) | *0.97* | *0.84-1.11* | *0.6182* |
| ≥66 | 92 (8.3) | 2035 (10.7) | 2127 (10.6) | *0.73* | *0.58-0.92* | *0.0069* |
| *Missing* | *26* | *426* | *452* |  |  |  |
| **Education** |  |  |  |  |  |  |
| First level | 107 (9.8) | 1132 (6.0) | 1239 (6.2) | *2.02* | *1.62-2.51* | *<.0001* |
| Second level | 502 (46.0) | 7321 (39.1) | 7823 (39.5) | *1.46* | *1.29-1.66* | *<.0001* |
| University | 482 (44.2) | 10282 (54.9) | 10764 (54.3) | *Reference* |  |  |
| *Missing* | *40* | *652* | *692* |  |  |  |
| **Work** |  |  |  |  |  |  |
| Workers | 739 (66.0) | 13338 (69.7) | 14077 (69.5) | *Reference* |  |  |
| Students | 76 (6.8) | 1437 (7.5) | 1513 (7.5) | *0.96* | *0.75-1.22* | *0.7068* |
| Unemployed | 72 (6.4) | 759 (4.0) | 831 (4.1) | *1.71* | *1.33-2.21* | *<.0001* |
| Others | 232 (20.7) | 3600 (18.8) | 3832 (18.9) | *1.16* | *1.00-1.35* | *0.0514* |
| *Missing* | *12* | *253* | *265* |  |  |  |
| **House** |  |  |  |  |  |  |
| 1-2 rooms | 208 (19.1) | 2949 (15.7) | 3157 (15.9) | *1.27* | *1.09-1.48* | *0.0029* |
| ≥3 rooms | 883 (80.9) | 15873 (84.3) | 16756 (84.1) | *Reference* |  |  |
| *Missing* | *40* | *565* | *605* |  |  |  |
| **Live with** |  |  |  |  |  |  |
| Alone | 151 (13.5) | 2618 (13.8) | 2769 (13.7) | *Reference* |  |  |
| 2 components | 273 (24.5) | 5335 (28.0) | 5608 (27.8) | *0.89* | *0.72-1.09* | *0.2507* |
| 3 components | 270 (24.2) | 4658 (24.5) | 4928 (24.5) | *1.01* | *0.82-1.23* | *0.9621* |
| More than 3 components | 421 (37.8) | 6420 (33.7) | 6841 (34.0) | *1.14* | *0.94-1.38* | *0.19* |
| *Missing* | *16* | *356* | *372* |  |  |  |
| **New health problems during previous14 days** |  |  |  |  |  |  |
| Nothing | 931 (84.7) | 17098 (91.2) | 18029 (90.8) | *Reference* |  |  |
| Symptomatics | 138 (12.6) | 1245 (6.6) | 1383 (7.0) | *2.04* | *1.69-2.46* | *<.0001* |
| Non symptomatics | 30 (2.7) | 410 (2.2) | 440 (2.2) | *1.34* | *0.92-1.96* | *0.1240* |
| *Missing* | *32* | *634* | *666* |  |  |  |
| **Contact with Covid-19 positive people** |  |  |  |  |  |  |
| Yes | 155 (13.9) | 1800 (9.4) | 1955 (9.7) | *1.55* | *1.30-1.85* | *<.0001* |
| No | 960 (86.1) | 17298 (90.6) | 18258 (90.3) | *Reference* |  |  |
| *Missing* | *16* | *289* | *305* |  |  |  |
| **Left the house in the previous week** |  |  |  |  |  |  |
| Yes | 586 (53.8) | 12097 (64.5) | 12683 (63.9) | *Reference* |  |  |
| No | 504 (46.2) | 6652 (35.5) | 7156 (36.1) | *1.56* | *1.38-1.77* | *<.0001* |
| *Missing* | *41* | *638* | *679* |  |  |  |
| **Total** | **1131 (5.5)** | **13017 (94.5)** | **20518 (100)** |  |  |  |

**Additional Table 5.** Results of logistic regression model for total responders (anxiety)

|  | **Model 1** | | | **Model 2** | | | **Model 3** | | |
| --- | --- | --- | --- | --- | --- | --- | --- | --- | --- |
|  | **OR** | **CI 95%** | **p** | **OR** | **CI 95%** | **p** | **OR**  **Weighted**  **for residence, gender, age and education** | **CI 95%** | **p** |
| **Gender** |  |  |  |  |  |  |  |  |  |
| Female *vs* Male | 2.27 | 1.92-2.68 | <.0001 | 2.20 | 1.83-2.65 | <.0001 | 2.64 | 2.24-3.11 | <.0001 |
| Missing *vs* Male | 1.81 | 1.22-2.70 | 0.0034 |  |  |  |  |  |  |
| **Age** |  |  |  |  |  |  |  |  |  |
| 18-30 *vs* 31-50 | 0.97 | 0.78-1.20 | 0.7563 | 1.02 | 0.80-1.30 | 0.8700 | 1.36 | 1.04-1.77 | 0.0246 |
| 51-65 *vs* 31-50 | 0.95 | 0.82-1.11 | 0.5327 | 0.93 | 0.79-1.10 | 0.4071 | 0.81 | 0.66-0.99 | 0.0428 |
| ≥66 *vs* 31-50 | 0.78 | 0.59-1.03 | 0.0851 | 0.81 | 0.58-1.12 | 0.2077 | 1.10 | 0.83-1.47 | 0.5072 |
| Missing *vs* 31-50 | 0.89 | 0.58-1.38 | 0.6151 |  |  |  |  |  |  |
| **Education** |  |  |  |  |  |  |  |  |  |
| First level *vs* University | 1.98 | 1.57-2.50 | <.0001 | 2.14 | 1.65-2.77 | <.0001 | 2.25 | 1.67-3.02 | <.0001 |
| Second level *vs* University | 1.47 | 1.28-1.68 | <.0001 | 1.53 | 1.32-1.78 | <.0001 | 1.52 | 1.20-1.92 | 0.0006 |
| Missing *vs* University | 1.31 | 0.93-1.86 | 0.1233 |  |  |  |  |  |  |
| **Work** |  |  |  |  |  |  |  |  |  |
| Student *vs* Workers | 0.86 | 0.64-1.16 | 0.3195 | 0.85 | 0.60-1.20 | 0.3551 | 0.44 | 0.31-0.63 | <.0001 |
| Unemployed *vs* Workers | 1.52 | 1.17-1.96 | 0.0016 | 1.39 | 1.03-1.87 | 0.0299 | 0.81 | 0.61-1.08 | 0.1481 |
| Other *vs* Workers | 1.16 | 0.96-1.40 | 0.1279 | 1.13 | 0.91-1.41 | 0.2678 | 0.74 | 0.59-0.92 | 0.0066 |
| Missing *vs* Workers | 0.78 | 0.43-1.42 | 0.4214 |  |  |  |  |  |  |
| **House** |  |  |  |  |  |  |  |  |  |
| 1-2 Rooms *vs* ≥3 Rooms | 1.29 | 1.08-1.53 | 0.0045 | 1.19 | 0.97-1.45 | 0.0887 | 1.45 | 1.19-1.75 | 0.0002 |
| Missing *vs* ≥3 Rooms | 1.38 | 0.98-1.95 | 0.0685 |  |  |  |  |  |  |
| **Live with** |  |  |  |  |  |  |  |  |  |
| 2 components *vs* Alone | 1.09 | 0.88-1.34 | 0.4276 | 1.13 | 0.89-1.43 | 0.3163 | 1.11 | 0.86-1.43 | 0.4069 |
| 3 components *vs* Alone | 1.13 | 0.95-1.35 | 0.1779 | 1.10 | 0.89-1.34 | 0.3776 | 1.62 | 1.30-2.01 | <.0001 |
| More than 3 components *vs* Alone | 1.27 | 1.08-1.51 | 0.0050 | 1.30 | 1.07-1.58 | 0.0068 | 1.72 | 1.39-2.12 | <.0001 |
| Missing *vs* Alone | 0.82 | 0.48-1.39 | 0.4604 |  |  |  |  |  |  |
| **New health problems during previous 14 days** |  |  |  |  |  |  |  |  |  |
| Symptomatic *vs* Nothing | 1.73 | 1.42-2.12 | <.0001 | 1.60 | 1.27-2.01 | <.0001 | 1.02 | 0.70-1.49 | 0.9181 |
| Non symptomatic *vs* Nothing | 1.08 | 0.73-1.60 | 0.7147 | 1.18 | 0.76-1.82 | 0.4636 | 2.77 | 1.91-4.02 | <.0001 |
| Missing *vs* Nothing | 0.86 | 0.59-1.25 | 0.4409 |  |  |  |  |  |  |
| **Contact with Covid-19 positive people** |  |  |  |  |  |  |  |  |  |
| Yes *vs* No | 1.37 | 1.13-1.66 | 0.0014 | 1.34 | 1.07-1.66 | 0.0091 | 1.03 | 0.74-1.45 | 0.8533 |
| Missing *vs* No | 0.93 | 0.56-1.55 | 0.7786 |  |  |  |  |  |  |
| **Left the house in the previous week** |  |  |  |  |  |  |  |  |  |
| No *vs* Yes | 1.40 | 1.23-1.59 | <.0001 | 1.42 | 1.23-1.64 | <.0001 | 1.92 | 1.66-2.23 | <.0001 |
| Missing *vs* Yes | 1.21 | 0.87-1.68 | 0.2623 |  |  |  |  |  |  |

Hosmer and Lemeshow Goodness of Fit test:

Model 1: χ^2^= 10.3404; df =8; p-value=0.2419

Model 2: χ^2^= 3.5208; df =8; p-value=0.8976

Model 3: χ^2^=49.4616; df =8; p-value=<0.0001

**Additional Table 6.** Characteristics of total responders by physical symptoms

|  | **Yes** | **No** | **Total** | **OR** | **CI 95%** | **p-value** |
| --- | --- | --- | --- | --- | --- | --- |
| **Gender** |  |  |  |  |  |  |
| Male | 678 (86.3) | 13264 (69.2) | 13942 (69.9) | *Reference* |  |  |
| Female | 108 (13.7) | 5899 (30.8) | 6007 (30.1) | *2.79* | *2.27-3.43* | *<.0001* |
| *Missing* | *16* | *553* | *569* |  |  |  |
| **Age** |  |  |  |  |  |  |
| 18-30 | 133 (17.1) | 2966 (15.4) | 3099 (15.4) | *0.94* | *0.77-1.15* | *0.5295* |
| 31-50 | 371 (47.7) | 7753 (40.2) | 8124 (40.5) | *Reference* |  |  |
| 51-65 | 229 (29.5) | 6487 (33.6) | 6716 (33.5) | *0.74* | *0.62-0.87* | *0.0004* |
| ≥66 | 44 (5.7) | 2083 (10.8) | 2127 (10.6) | *0.44* | *0.32-0.61* | *<.0001* |
| *Missing* | *25* | *427* | *452* |  |  |  |
| **Education** |  |  |  |  |  |  |
| First level | 63 (8.1) | 1176 (6.2) | 1239 (6.2) | *1.50* | *1.14-1.97* | *0.0038* |
| Second level | 339 (43.8) | 7484 (39.3) | 7823 (39.5) | *1.27* | *1.09-1.47* | *0.0021* |
| University | 372 (48.1) | 10392 (54.5) | 10764 (54.3) | *Reference* |  |  |
| *Missing* | *28* | *664* | *692* |  |  |  |
| **Work** |  |  |  |  |  |  |
| Workers | 559 (70.7) | 13518 (69.5) | 14077 (69.5) | *Reference* |  |  |
| Students | 66 (8.3) | 1447 (7.4) | 1513 (7.5) | *1.10* | *0.85-1.43* | *0.4613* |
| Unemployed | 55 (7.0) | 776 (4.0) | 831 (4.1) | *1.71* | *1.29-2.28* | *0.0002* |
| Others | 111 (14.0) | 3721 (19.1) | 3832 (18.9) | *0.72* | *0.59-0.89* | *0.0020* |
| *Missing* | *11* | *254* | *265* |  |  |  |
| **House** |  |  |  |  |  |  |
| 1-2 rooms | 150 (19.2) | 3007 (15.7) | 3157 (15.9) | *1.27* | *1.06-1.53* | *0.0099* |
| ≥3 rooms | 633 (80.8) | 16123 (84.3) | 16756 (84.1) | *Reference* |  |  |
| *Missing* | *19* | *586* | *605* |  |  |  |
| **Live with** |  |  |  |  |  |  |
| Alone | 90 (11.5) | 2679 (13.8) | 2769 (13.7) | *Reference* |  |  |
| 2 components | 207 (26.5) | 5401 (27.9) | 5608 (27.8) | *1.14* | *0.89-1.47* | *0.3050* |
| 3 components | 182 (23.3) | 4746 (24.5) | 4928 (24.5) | *1.14* | *0.88-1.48* | *0.3128* |
| More than 3 components | 301 (38.6) | 6540 (33.8) | 6841 (34.0) | *1.37* | *1.08-1.74* | *0.0101* |
| *Missing* | *22* | *350* | *372* |  |  |  |
| **New health problems during previous 14 days** |  |  |  |  |  |  |
| Nothing | 613 (79.7) | 17416 (91.3) | 18029 (90.8) | *Reference* |  |  |
| Symptomatics | 128 (16.6) | 1255 (6.6) | 1383 (7.0) | *2.90* | *2.38-3.54* | *<.0001* |
| Non symptomatics | 28 (3.6) | 412 (2.2) | 440 (2.2) | *1.93* | *1.31-2.86* | *0.0010* |
| *Missing* | *33* | *633* | *666* |  |  |  |
| **Contact with Covid-19 positive people** |  |  |  |  |  |  |
| Yes | 122 (15.5) | 1833 (9.4) | 1955 (9.7) | *1.77* | *1.45-2.16* | *<.0001* |
| No | 663 (84.5) | 17595 (90.6) | 18258 (90.3) | *Reference* |  |  |
| *Missing* | *17* | *288* | *305* |  |  |  |
| **Left the house in the previous week** |  |  |  |  |  |  |
| Yes | 405 (52.0) | 12278 (64.4) | 12683 (63.9) | *Reference* |  |  |
| No | 374 (48.0) | 6782 (35.6) | 7156 (36.1) | *1.67* | *1.45-1.93* | *<.0001* |
| *Missing* | *23* | *656* | *679* |  |  |  |
| **Total** | **802 (3.9)** | **19716 (96.1)** | **20518 (100)** |  |  |  |

**Additional Table 7.** Results of the logistic regression model for total responders (physical symptoms)

|  | **Model 1** | | | **Model 2** | | | **Model 3** | | |
| --- | --- | --- | --- | --- | --- | --- | --- | --- | --- |
|  | **OR** | **CI 95%** | **p** | **OR** | **CI 95%** | **p** | **OR**  **Weighted**  **for residence, gender, age and education** | **CI 95%** | **p** |
| **Gender** |  |  |  |  |  |  |  |  |  |
| Female *vs* Male | 2.70 | 2.19-3.32 | <.0001 | 2.54 | 2.02-3.19 | <.0001 | 2.26 | 1.87-2.73 | <.0001 |
| Missing *vs* Male | 1.43 | 0.83-2.46 | 0.1928 |  |  |  |  |  |  |
| **Age** |  |  |  |  |  |  |  |  |  |
| 18-30 *vs* 31-50 | 0.98 | 0.77-1.25 | 0.8811 | 1.04 | 0.80-1.37 | 0.7531 | 1.57 | 1.18-2.09 | 0.0019 |
| 51-65 *vs* 31-50 | 0.77 | 0.64-0.92 | 0.0042 | 0.82 | 0.67-1.00 | 0.0501 | 0.68 | 0.53-0.86 | 0.0013 |
| ≥66 *vs* 31-50 | 0.63 | 0.43-0.92 | 0.0173 | 0.63 | 0.40-0.99 | 0.0444 | 0.54 | 0.38-0.78 | 0.0011 |
| Missing *vs* 31-50 | 1.00 | 0.64-1.58 | 0.9839 |  |  |  |  |  |  |
| **Education** |  |  |  |  |  |  |  |  |  |
| First level *vs* University | 1.74 | 1.31-2.33 | 0.0002 | 1.88 | 1.37-2.60 | 0.0001 | 1.71 | 1.20-2.44 | 0.0028 |
| Second level *vs* University | 1.36 | 1.16-1.59 | 0.0001 | 1.36 | 1.14-1.62 | 0.0006 | 1.42 | 1.09-1.86 | 0.0092 |
| Missing *vs* University | 1.20 | 0.80-1.81 | 0.3797 |  |  |  |  |  |  |
| **Work** |  |  |  |  |  |  |  |  |  |
| Student *vs* Workers | 0.9 | 0.65-1.25 | 0.5233 | 0.84 | 0.57-1.24 | 0.3784 | 0.40 | 0.27-0.60 | <.0001 |
| Unemployed *vs* Workers | 1.530 | 1.14-2.05 | 0.0046 | 1.48 | 1.06-2.06 | 0.0205 | 1.03 | 0.76-1.41 | 0.8381 |
| Other *vs* Workers | 0.80 | 0.62-1.03 | 0.0905 | 0.78 | 0.59-1.05 | 0.0989 | 0.97 | 0.74-1.27 | 0.8330 |
| Missing *vs* Workers | 0.99 | 0.53-1.84 | 0.9726 |  |  |  |  |  |  |
| **House** |  |  |  |  |  |  |  |  |  |
| 1-2 Rooms *vs* ≥3 Rooms | 1.22 | 1.00-1.50 | 0.0513 | 1.13 | 0.89-1.43 | 0.3096 | 1.10 | 0.87-1.40 | 0.4153 |
| Missing *vs* ≥3 Rooms | 0.76 | 0.47-1.24 | 0.2765 |  |  |  |  |  |  |
| **Live with** |  |  |  |  |  |  |  |  |  |
| 2 components *vs* Alone | 0.85 | 0.66-1.10 | 0.2203 | 0.89 | 0.66-1.18 | 0.4151 | 0.90 | 0.66-1.23 | 0.5252 |
| 3 components *vs* Alone | 0.92 | 0.75-1.14 | 0.4590 | 0.89 | 0.70-1.12 | 0.3209 | 1.14 | 0.89-1.47 | 0.3083 |
| More than 3 components *vs* Alone | 1.05 | 0.86-1.27 | 0.6466 | 1.05 | 0.84-1.30 | 0.6794 | 1.35 | 1.06-1.71 | 0.0139 |
| Missing *vs* Alone | 1.62 | 1.01-2.58 | 0.0432 |  |  |  |  |  |  |
| **New health problems during previous 14 days** |  |  |  |  |  |  |  |  |  |
| Symptomatic *vs* Nothing | 2.41 | 1.95-2.99 | <.0001 | 2.27 | 1.78-2.90 | <.0001 | 2.40 | 1.73-3.35 | <.0001 |
| Non symptomatic *vs* Nothing | 1.50 | 0.99-2.27 | 0.0540 | 1.37 | 0.84-2.23 | 0.2015 | 3.56 | 2.36-5.37 | <.0001 |
| Missing *vs* Nothing | 1.40 | 0.96-2.04 | 0.0779 |  |  |  |  |  |  |
| **Contact with Covid-19 positive people** |  |  |  |  |  |  |  |  |  |
| Yes *vs* No | 1.32 | 1.06-1.64 | 0.0123 | 1.32 | 1.03-1.69 | 0.0263 | 0.78 | 0.52-1.18 | 0.2386 |
| Missing *vs* No | 1.56 | 0.94-2.58 | 0.0832 |  |  |  |  |  |  |
| **Left the house in the previous week** |  |  |  |  |  |  |  |  |  |
| No *vs* Yes | 1.41 | 1.21-1.64 | <.0001 | 1.39 | 1.17-1.65 | 0.0001 | 1.53 | 1.29-1.82 |  |
| Missing *vs* Yes | 1.00 | 0.65-1.55 | 0.9910 |  |  |  |  |  |  |

Hosmer and Lemeshow Goodness of Fit test:

Model 1: χ^2^= 2.8041; df =8; p-value=0.9460

Model 2: χ^2^= 11.4871; df =8; p-value=0.1756

Model 3: χ^2^=28.5550; df =8; p-value=0.0004
